# Dual Focus-3D: A Hybrid Deep Learning Approach for Robust 3D Gaze Estimation

**DOI:** 10.3390/s25134086

**Published:** 2025-06-30

**Authors:** Abderrahmen Bendimered, Rabah Iguernaissi, Mohamad Motasem Nawaf, Rim Cherif, Séverine Dubuisson, Djamal Merad

**Affiliations:** Laboratoire d’Informatique et des Systèmes, CNRS UMR 7020, Aix-Marseille University, 13009 Marseille, France; rabah.iguernaissi@lis-lab.fr (R.I.); motasem.nawaf@lis-lab.fr (M.M.N.); rim.cherif@lis-lab.fr (R.C.); severine.dubuisson@lis-lab.fr (S.D.); djamal.merad@lis-lab.fr (D.M.)

**Keywords:** 3D gaze estimation, computer vision, multimodal fusion, EyeLis dataset

## Abstract

Estimating gaze direction is a key task in computer vision, especially for understanding where a person is focusing their attention. It is essential for applications in assistive technology, medical diagnostics, virtual environments, and human–computer interaction. In this work, we introduce Dual Focus-3D, a novel hybrid deep learning architecture that combines appearance-based features from eye images with 3D head orientation data. This fusion enhances the model’s prediction accuracy and robustness, particularly in challenging natural environments. To support training and evaluation, we present EyeLis, a new dataset containing 5206 annotated samples with corresponding 3D gaze and head pose information. Our model achieves state-of-the-art performance, with a MAE of 1.64° on EyeLis, demonstrating its ability to generalize effectively across both synthetic and real datasets. Key innovations include a multimodal feature fusion strategy, an angular loss function optimized for 3D gaze prediction, and regularization techniques to mitigate overfitting. Our results show that including 3D spatial information directly in the learning process significantly improves accuracy.

## 1. Introduction

Gaze direction is a critical source of information for assessing individuals’ intentions and behaviors; it allows for the real-time inference of human intent, particularly regarding attention and engagement. For example, observing an individual’s gaze direction can provide valuable insights into their level of fatigue [1], support gaze prediction in contexts such as driving [2,3], or, in the medical domain, help to evaluate levels of intentionality in patients with neurological or other disorders [4]. Moreover, beyond the analysis of spontaneous human behaviors, gaze estimation also plays a significant role in controlled interaction settings, such as wheelchair navigation [5], activation of mechatronic systems [6], and medical robotics, notably in the control of robotic arms [7].

In some applications, gaze-related information is simultaneously essential for interpreting spontaneous intention and for enabling action control; for example, in immersive environments such as VR, the use of gaze-based interaction techniques offers a significant enhancement of user–environment engagement [8,9]. Despite the broad range of applications, accurately predicting gaze direction remains a fundamental and persistent challenge. One of the primary difficulties lies in the variability and complexity of the natural environment. Gaze estimation systems must operate reliably under diverse lighting environments, across a wide range of head poses, and in the presence of partial occlusions, such as eyeglasses, eyelashes, or motion blur. These factors can significantly degrade the precision of gaze prediction models.

Broadly, existing gaze estimation methods can be divided into two main categories [10]: model-based approaches, which rely on geometric modeling of eye anatomy, and appearance-based approaches, which directly learn mappings from facial or eye images using machine learning or deep learning techniques. While model-based methods typically achieve high accuracy under controlled conditions, they require specialized equipment and are sensitive to lighting and head pose variations. Appearance-based methods, on the other hand, offer greater flexibility but often struggle to capture complex three-dimensional head–gaze relationships and remain vulnerable to occlusions.

To overcome these limitations, hybrid approaches have emerged, combining geometric modeling with appearance-based learning to enhance robustness and generalization. Recent work, such as [11], demonstrates that integrating geometric constraints into learning pipelines can significantly improve gaze estimation performance.

However, several significant challenges remain. The first is the limited diversity of available data. Many existing datasets, such as Columbia Gaze [12] and UT Multiview Gaze Dataset [13], have limited variability in terms of lighting or extreme head poses, which reduce the models’ ability to generalize and adapt to diverse natural environments. Another major issue is the lack of precise 3D information in existing datasets. While some datasets include 3D annotations—such as the gaze target position or inferred gaze based on fixed target points—they often do not provide an exact 3D gaze direction vector within a metric reference frame. As a result, the actual gaze direction is not explicitly represented with the accuracy required for fine-grained 3D estimation. In this context, other authors in [14] proposed a 3D gaze estimation method based on a four-camera tracking system.

To address these limitations, combining eye images with head orientation vectors along with precise 3D annotations in a hybrid model appears to be a promising solution. Integrating these two sources of information could lead to a more reliable and robust estimation of gaze direction, even in uncontrolled conditions.

In this work, we propose an innovative approach for gaze direction estimation by leveraging 3D information. Our method combines a geometric model-based approach with an appearance-based approach, integrating CNN and MLP to extract eye visual features and incorporate head orientation data. Specifically, our model uses eye images together with a 3D head orientation vector to predict gaze direction within a metric reference frame. This approach ensures accurate gaze estimation without the need for invasive equipment to capture 3D head orientations or expensive techniques to detect the ocular region, thus simplifying and accelerating the estimation process.

We introduce the EyeLis dataset [15], which includes annotated images of eyes, as well as head orientation and gaze direction data in 3D. The annotations were performed using the methodology described in [16], which relies on non-invasive gaze estimation within a metric reference frame, with a tolerance of 2° for annotation errors. This dataset contains a total of 5206 annotated images and provides a valuable resource for the development and evaluation of 3D gaze estimation models under diverse and natural environment.

In summary, the main contributions of this paper are as follows: (a) a hybrid CNN-MLP architecture that combines visual and orientation data for 3D gaze estimation; (b) the demonstration of improved robustness and accuracy through 3D integration; and (c) the public release of the EyeLis dataset represents a valuable contribution to the community. Experimental results on EyeLis, as well as on other public datasets such as MPIIFaceGaze [17], RT-GENE [18], and UnityEye [19], demonstrate that our approach outperforms existing methods, achieving average angular errors of 1.64° for EyeLis, 3.24° for MPIIFaceGaze, 1.73° for RT-GENE, and 2.39° for UnityEye. These results validate the effectiveness of our method and pave the way for new applications in fields like AR, healthcare, and contactless human–computer interaction, while ensuring high accuracy.

The rest of the paper is organized as follows: Section 2 presents a review of the related works in the field. Section 3 details the EyeLis dataset and its creation process. Section 4 presents the proposed Dual Focus-3D methodology, including architecture and training. In Section 5, we provide an analysis of the experimental results, including the performance of the proposed method on several benchmark datasets. Finally, Section 6 discusses the limitations and practical considerations, as well as future research directions. Section 7 concludes the paper.

## 2. Related Work

Gaze estimation has attracted considerable research interest due to its wide range of applications in fields such as human–computer interaction, autonomous systems, and medical diagnostics. Early methods primarily relied on precise 3D geometric models of the eye to estimate gaze direction. However, with the advancement of machine learning, the focus has progressively shifted toward appearance-based techniques, particularly deep learning models, which have demonstrated significant improvements in robustness and adaptability to complex, unconstrained environments.

More recently, hybrid approaches combining geometric modeling with appearance-based learning have emerged, aiming to leverage the strengths of both strategies to enhance accuracy, generalization, and usability across diverse natural environment. In the following, we review the main categories of gaze estimation methods—model-based, appearance-based, and hybrid techniques—as well as the key datasets that have driven progress in this area.

### 2.1. Model-Based Methods

Model-based gaze estimation methods aim to predict gaze direction by relying on the geometric modeling of the eye. Early approaches typically used 2D anatomical features extracted from images, such as the center of the pupil or the boundary of the iris, to infer gaze direction. These methods assumed simplified geometric relationships between visible eye landmarks and gaze vectors, often estimating the point of regard based on pupil center displacement or corneal reflection patterns. For example, certain systems extracted these features and related them to screen coordinates or simple angular displacements [20]. While such 2D model-based methods offered good performance in controlled laboratory conditions, they faced notable limitations. They generally required specialized equipment such as high-resolution cameras or infrared illumination [21] to enhance feature visibility, and their accuracy degraded significantly in the presence of lighting variations, eyelid occlusions, or head pose changes.

To overcome these limitations, more recent model-based approaches introduced the construction of full 3D eye models. In these methods, a three-dimensional geometric representation of the eyeball is created, allowing for a more direct and interpretable mapping between eye features and gaze direction. Typically, these techniques detect key eye landmarks in 2D images, such as the pupil center and iris contour, and align them with a calibrated 3D model of the eye to reconstruct the 3D gaze vector in world coordinates [22,23]. The use of a 3D model provides greater robustness to variations in individual eye anatomy, as well as improved tolerance to small lighting changes and minor occlusions. Moreover, it allows the estimation of gaze not only on a 2D plane but within the full three-dimensional space, which is essential for many real-world applications such as robotics or AR.

However, 3D model-based methods are not without challenges. Their performance is highly dependent on the accuracy of the modeled eye parameters (e.g., eyeball radius, corneal curvature), which may vary significantly across individuals. Inaccurate modeling can introduce systematic errors. Additionally, they often require precise calibration procedures or controlled acquisition settings to ensure reliable landmark detection and model fitting. In environments with uncontrolled lighting, head motion, or partial occlusions, feature extraction can become unreliable, leading to degraded gaze estimation. Furthermore, methods relying on structured light patterns to detect corneal reflections can be perceived as intrusive or uncomfortable for users, limiting their usability in naturalistic settings.

In summary, model-based methods, evolving from simple 2D geometric assumptions to sophisticated 3D reconstructions, have demonstrated strong theoretical appeal and physical interpretability. Nevertheless, their practical application remains constrained by the complexity of accurately modeling and capturing individual eye characteristics under diverse natural environment.

### 2.2. Appearance-Based Methods

Appearance-based methods for gaze estimation aim to learn a direct mapping between visual inputs—typically facial or eye images—and gaze direction, without explicitly modeling anatomical features. Early approaches used classical machine learning techniques, such as SVR and GPR [24], relying on handcrafted features extracted from eye regions.

The emergence of deep learning profoundly transformed this field. CNNs became the dominant paradigm, thanks to their ability to automatically extract hierarchical visual features. One of the first applications of CNNs to gaze estimation was proposed by [25], who adapted a simplified LeNet architecture [26] to process single-channel eye images and directly infer gaze direction. By introducing dynamic weighting mechanisms, their network could prioritize the most informative regions of the input, enhancing prediction robustness.

Later work developed more sophisticated CNN-based frameworks. For instance, the Pictorial Gaze model introduced in [27] was an intermediate representation, termed “Gazemap”, capturing the spatial relationship between the eyeball and iris. The system performed a two-stage regression: first estimating the Gazemap from the input image, then predicting the gaze vector from this abstract representation. To better handle extreme head poses and lighting variability, asymmetric regression strategies were also explored, assuming that gaze directions between the two eyes remain correlated despite appearance differences [28].

Other studies have explored multi-stream architectures to integrate richer information. For example, the MAFI-Gaze model [29] compared single-stream approaches—using either head or eye images separately—to multi-stream models combining both modalities. The results highlighted the benefit of incorporating multiple visual cues to enhance gaze estimation, particularly under varied conditions.

While CNNs excel with high-resolution inputs, their performance degrades significantly for low-resolution images due to lost texture details. To address this, [30] proposed FSKT-GE, a feature-similarity knowledge transfer framework that aligns intermediate representations between high- and low-resolution networks via cosine similarity. By avoiding explicit super-resolution and instead enforcing distributional consistency, their method achieved state-of-the-art results on downsampled images from Gaze360 and RT-Gene (6.73°–13.61° mean angular error), demonstrating the viability of knowledge transfer for low-resolution gaze estimation.

Most CNN-based methods tend to focus on localized features without explicitly modeling head pose variations. Furthermore, appearance-based systems are sensitive to occlusions, such as those caused by eyeglasses, eyelashes, or shadows. Techniques such as radial symmetry detection for the pupil center have been proposed to mitigate these issues [31], but robustness remains a significant concern, especially in unconstrained real-world settings.

Beyond static image-based models, researchers have explored temporal modeling to capture dynamic aspects of gaze behavior. RNNs, particularly LSTM networks [32,33], have been employed to model sequential eye movement patterns over time. More recently, Transformer architectures [34,35] have been introduced, offering the ability to simultaneously model spatial and temporal dependencies, thus improving performance under highly variable conditions.

The success of appearance-based methods is closely tied to the availability of large and diverse annotated datasets. Early datasets such as EyeDiap [36] combined controlled and spontaneous gaze behaviors with 3D head pose annotations, while GazeCapture [37] enabled large-scale data collection in unconstrained mobile environments. More recent datasets like ETH-XGaze [38] introduced significant variations in head pose and gaze direction, further promoting generalization across natural environment.

Building on these advances, hybrid models have emerged, combining different network types to leverage complementary strengths. For instance, Gaze-Swin [39] integrates CNN-based feature extraction with Swin Transformer modules to simultaneously capture local details and global context, leading to further improvements in gaze estimation accuracy and robustness.

## 3. Gaze Dataset Generation

One of the main challenges in gaze estimation is the scarcity of highly accurate annotated data, particularly for gaze direction. To address this, we utilized the EyeLis-DB database, which was developed using a novel, non-intrusive method to simultaneously capture gaze direction and head orientation via the Kinect Azure camera in real-world 3D environments. The database was constructed from real-time facial recordings under a fixed multi-target setup, with orientation vectors computed per frame using over 83,000 annotated frames collected at 30 FPS from 8 participants. The full details of the acquisition protocol, annotation procedure, and dataset characteristics are described in [16], and all images are provided at a resolution of 960×540 pixels.

The EyeLis-DB database enables users to select a predefined annotation error threshold when generating samples, where each image is annotated based on real-world data and validated against 3D gaze target positions. The annotations include the error between the actual and labeled gaze directions, offering a flexible process for generating datasets with controlled accuracy levels. This makes it possible to train and evaluate gaze estimation models under varying error conditions.

In our work, we selected a threshold of 2° and derived the EyeLis dataset accordingly. This curated subset ensures high annotation accuracy and was used in all experiments presented in this paper.

## 4. Proposed Methodology

**Figure 1:**
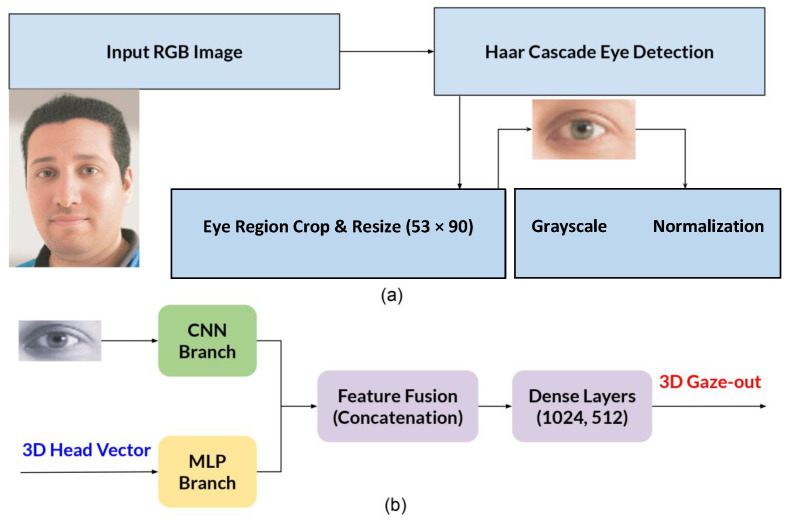
Overview of the proposed 3D gaze estimation pipeline. (**a**) Eye detection and preprocessing using Haar cascade and OpenCV. (**b**) Dual-branch architecture with CNN and MLP for feature extraction and gaze prediction.

Our gaze estimation framework (Figure 1) uses a dual-branch architecture combining a CNN and an MLP for accurate 3D gaze prediction. As shown in Figure 1a, input RGB images are first processed using a Haar cascade-based method [40] with OpenCV’s version 4.11.0 pre-trained eye detector [41], which has shown reliable performance in real-time applications [42].

Figure 1b shows the architecture of the proposed system, which includes two parallel branches: a residual CNN for visual feature extraction from grayscale eye images, with normalization and resizing to 53×90 pixels, and an MLP for processing 3D head orientation vectors. The extracted features are concatenated and refined through dense layers with dropout regularization.

**Figure 2:**
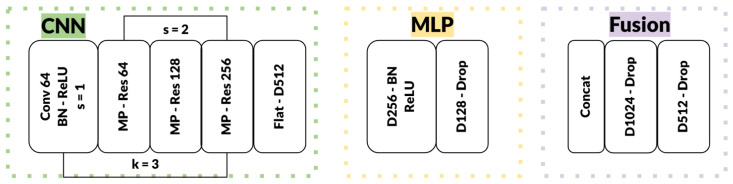
Dual Focus-3D feature fusion module.

Figure 2 provides a detailed breakdown of this architecture; the CNN branch includes successive residual blocks with increasing channel sizes (64→128→256), each followed by max-pooling layers. The MLP branch consists of fully connected layers with ReLU activations. The final fused features are processed through dense layers (1024→512 units), effectively fusing spatial and positional information for robust gaze vector regression. This hybrid design achieves computational efficiency without sacrificing accuracy, as the residual connections enhance feature propagation while dropout prevents overfitting, making the system suitable for real-time applications in human–computer interaction.

### 4.1. Eye Feature Extraction

The process begins with an initial convolution applied to a grayscale images. This image is then processed using a CNN. The first step involves a convolution layer (Conv2D-64) that extracts edges and textures from the image. This operation is defined by the following equation:

(1) $$F_{0}=\mathrm{ReLU}\left(W_{0}*I+b_{0}\right)$$

where $F_{0}$ is the output of the first convolutional layer, $W_{0}$ is the convolution filter, *I* is the input image (eye patch), and $b_{0}$ is the bias.

Next, a max-pooling operation is applied to reduce the spatial dimensions of the image by a factor of 2. Feature extraction then continues through three hierarchical residual blocks with 64, 128, and 256 channels, respectively. These blocks capture intermediate and high-level features. Each block is defined as

(2) $$F_{l+1}=\mathrm{ReLU}\left(F_{l}+H\left(F_{l},W_{l}\right)\right)$$

where $F_{l}$ is the input to the residual block, $H(F_{l},W_{l})$ represents a transformation function consisting of two convolutional layers followed by batch normalization, and $F_{l+1}$ is the output. These residual connections help to mitigate the vanishing gradient problem.

The extracted features are then flattened and projected into a 512-dimensional space as follows:

(3) $$f_{eye}=W_{dense}\cdot \mathrm{Flatten}\left(F_{3}\right)+b_{dense}$$

where $f_{eye}$ is the eye feature vector, $W_{dense}$ and $b_{dense}$ are the weights and bias of the dense layer, and $\mathrm{Flatten}(F_{3})$ denotes the flattened output from the third residual block.

The goal of this step is to produce a compact feature vector from the eye image. This approach supports real-time processing by avoiding the complexity and computational overhead associated with multi-head attention mechanisms used in previous works, as discussed in Section 1.

### 4.2. Head Pose Feature Extraction

Head pose feature extraction starts with a 3D vector representing head orientation (yaw, pitch, roll). This vector is normalized and used as input to the model. It is then non-linearly projected through two dense layers, defined as

(4) $$f_{head}=W_{2}\cdot \mathrm{ReLU}\left(W_{1}\cdot h+b_{1}\right)+b_{2}$$

where $f_{head}$ is the head feature vector, *h* is the input head pose vector, $W_{1}\in R^{256\times 3}$, $W_{2}\in R^{128\times 256}$, and $b_{1}$, $b_{2}$ are the corresponding biases. Head pose input *h* helps resolve gaze direction ambiguity.

### 4.3. Feature Fusion

The features extracted from the eyes and head are concatenated into a single vector:

(5) $$\left[f_{eye};f_{head}\right]\in R^{640}$$

This fused vector combines information from both modalities. A joint representation is then learned through a dense layer:

(6) $$z=\mathrm{ReLU}(W_{f}\left[f_{eye};f_{head}\right]+b_{f})$$

where $W_{f}\in R^{1024\times 640}$ and $b_{f}$ is the bias term. A dropout with probability p=0.5 is applied for regularization. Alignment is implicitly performed using a dot product:

(7) $$g=f_{eye}^{T}Mf_{head}$$

where *g* is the gaze–head alignment score, and *M* is a learned metric space matrix.

### 4.4. Gaze Regression Head

The model returns a 3D vector representing the gaze direction in space, computed as follows:

(8) $$\hat{g}=W_{o}\cdot z+b_{o}$$

where $\hat{g}$ is the predicted gaze vector, and $W_{o}$, $b_{o}$ are the weights and bias of the final dense layer.

The loss function used to train the model minimizes the angular error between the true gaze vector $g_{i}$ and the predicted one ${\hat{g}}_{i}$:

(9) $$L=\frac{1}{N}\sum_{i=1}^{N}\cos^{-1}\left(\frac{g_{i}\cdot {\hat{g}}_{i}}{∥g_{i}∥∥{\hat{g}}_{i}∥}\right)$$

This loss function allows for precise prediction of gaze direction in 3D space, which is crucial for 3D gaze estimation tasks where only the vector’s orientation is important.

## 5. Experiments

In this section, we present the experimental setup and evaluation of the proposed Dual Focus-3Dmodel. We describe the datasets used, detail the training protocols, and report quantitative and qualitative results under various evaluation scenarios. These include both intra-dataset and cross-dataset experiments, designed to assess the model’s accuracy, robustness, and generalization across diverse conditions.

### 5.1. Datasets

As shown in Table 1, the datasets used differ in terms of acquisition hardware, lighting conditions, and participant count.

MPIIFaceGaze comprises 213,659 images collected from 15 individuals using a laptop webcam in everyday settings over an extended period. The data covers a wide range of illumination scenarios and does not impose constraints on head orientation. Among the full dataset, 37,667 images are annotated with accurate gaze vectors, pupil locations, and facial landmarks, providing a solid base for supervised learning tasks in gaze estimation.

RT-GENE includes multimodal recordings acquired with a Kinect v2 sensor combined with a motion capture system. It contains data from 15 participants recorded over 17 sessions. The dataset offers synchronized RGB, depth, and infrared streams along with head pose and gaze direction annotations. Data was collected in naturally lit indoor environments, enabling diverse head orientations and natural conditions.

UnityEye is a synthetic dataset generated using a 3D graphics engine, simulating eye images with precise control over pose, lighting, and gaze direction. It supports the creation of unlimited samples with detailed ground truth. For our experiments, we generated a custom subset of 66,316 synthetic images, facilitating model pre-training and evaluation under fully controlled conditions.

The EyeLis-DB was recorded by the Azure Kinect v3 sensor in a lab environment. It includes recordings from 8 participants, each one captured in 5 predefined head positions, resulting in 40 total sessions. Sessions were conducted under both natural daylight and uniform artificial lighting, introducing a mix of conditions to evaluate model robustness. The dataset is designed to provide consistent pose variation while maintaining real-world variability in lighting.

### 5.2. Experimental Results Analysis

The model was developed using the TensorFlow framework version 2.19.0, utilizing the Adam optimizer with an initial learning rate of 0.0005 and a batch size of 32. The training process was set for 100 epochs. Early stopping was applied to optimize training, monitoring the validation loss and halting when no improvement was observed over 10 consecutive epochs. Additionally, the learning rate was reduced to 0.0001 after 5 epochs without improvement.

To prevent overfitting, a 50% dropout rate was applied, especially in the fully connected layers. A custom angular loss function was employed to minimize the angular error between the predicted and actual gaze vectors.

Our model, trained on a custom dataset with 70% for training and 30% for testing, achieves a MAE of 1.64° on the EyeLis dataset.

In Table 2, we evaluate the model on the MPIIFaceGaze dataset, where it achieves a MAE of 3.24°, showcasing the model’s effectiveness in controlled environments with artificial lighting. In Table 3, the RT-GENE dataset yields a MAE of 1.73°, demonstrating that our model can handle natural lighting conditions with high precision. These results confirm that our architecture generalizes well across different datasets and ensures that the integration of 3D features significantly enhances the training process, improving accuracy and robustness. The architecture also includes a regression component that can detect head pose variations, even when trained on different datasets.

Table 4 presents results on the UnityEye dataset, with a MAE of 2.39°, evaluated on a synthetic dataset. This helps confirm that our model is resistant to overfitting, and it consistently performs well even on synthetic data, further validating its generalizability.

These tables summarize the performance of the Dual Focus-3D model on the MPIIFaceGaze, RT-GENE, and UnityEye datasets, demonstrating its ability to achieve accurate gaze estimation across various conditions.

Each table includes baseline methods that were specifically developed and evaluated on the corresponding dataset, to ensure a fair and meaningful comparison.

As observed, the Dual Focus-3D approach outperforms other state-of-the-art methods by providing more accurate gaze estimations while maintaining a compact and efficient architecture. This makes our model a reliable and precise solution for gaze tracking applications.

**Figure 3:**
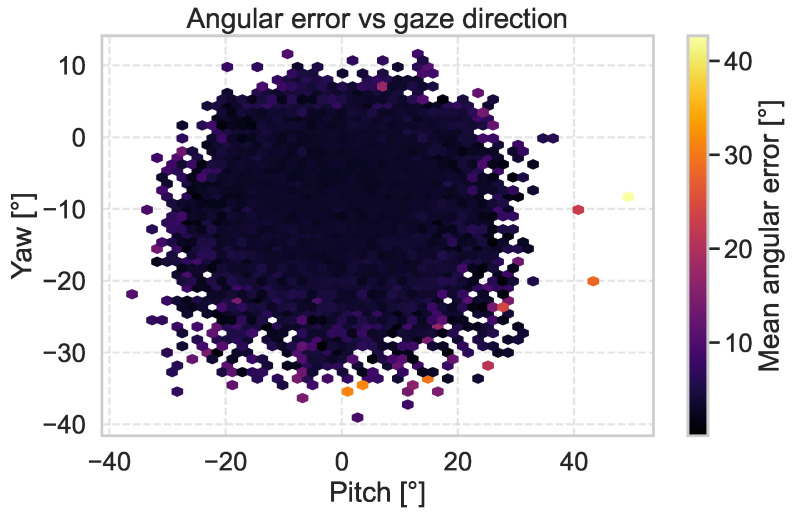
MAE heatmap across gaze directions on the MPIIFaceGaze test set.

Figure 3 shows a 2D heatmap of the MAE relative to the ground-truth gaze direction, represented in spherical coordinates (pitch and yaw), on the MPIIFaceGaze dataset. The heatmap reveals low prediction errors across the gaze space, indicating that the model achieves high accuracy, even for extreme gaze directions.

**Figure A1:**
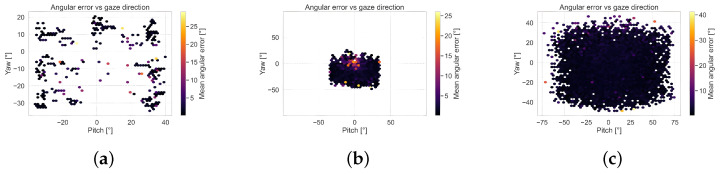
MAE heatmap across gaze directions on the (**a**) EyeLis, (**b**) RT-GENE, and (**c**) UnityEye test sets.

The results confirm that, across the EyeLis, UnityEye, and RT-GENE datasets as shown in Figure A1, the model accurately predicts gaze direction even when the gaze direction is extreme.

**Figure 4:**
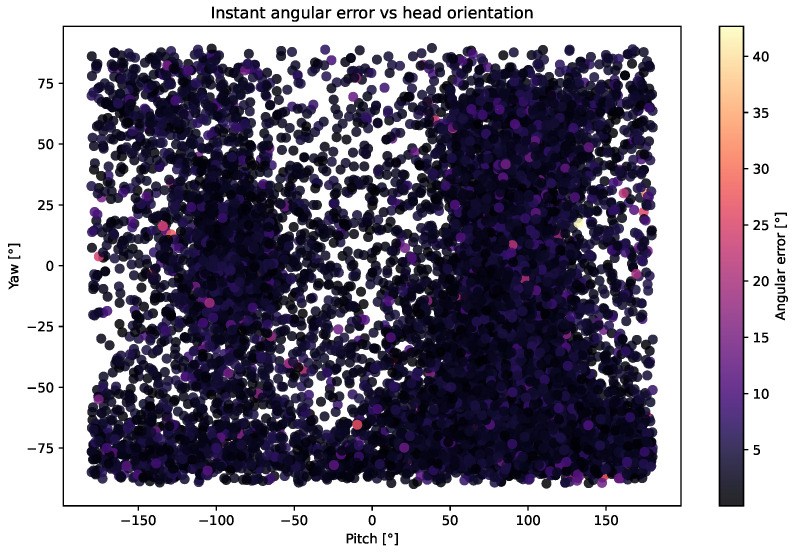
MAE distribution relative to head orientation evaluated on the MPIIFaceGaze test set.

Figure 4 shows the distribution of the MAE as a function of gaze direction using the MPIIFaceGaze dataset.

**Figure A2:**
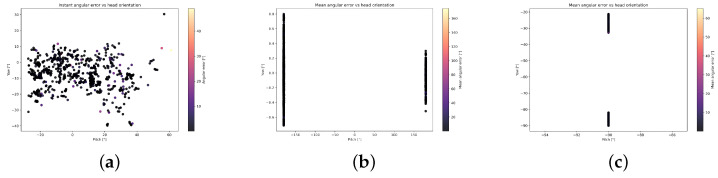
Angular error distribution across head–gaze angle on (**a**) EyeLis, (**b**) RT-GENE, and (**c**) Unity datasets.

Analyzing the results in Figure A2 for the EyeLis, RT-GENE, and UnityEye datasets shows that the model accurately predicts gaze direction, even when the head orientation is extreme.

**Figure 5:**
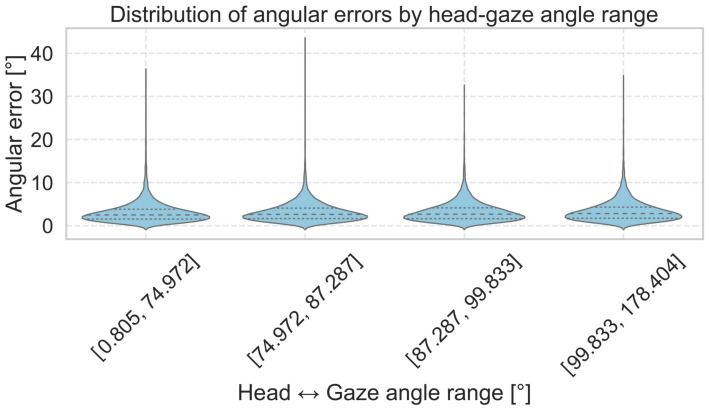
Angular error distribution across head–gaze angle quantiles on theMPIIFaceGaze test set.

**Figure A3:**
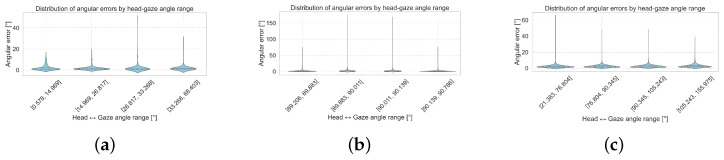
Angular error distribution across head–gaze angle quantiles for (**a**) EyeLis, (**b**) RT-GENE, and (**c**) UnityEye datasets.

Figure 5 and Figure A3 show the distribution of angular errors relative to the angle between the head orientation vector and the ground-truth gaze direction for the MPIIFaceGaze, EyeLis, RT-GENE and UnityEye datasets. The horizontal axis represents the angle between the head and gaze, divided into four quantile-based bins, while the vertical axis shows the corresponding angular error in degrees.

We observe that the predictions of our Dual Focus-3D model remain consistent across different head–gaze angles. The values for mean and median angular errors are nearly identical across the four bins, showing that the model is well-trained and effectively predicts the true gaze direction based on the image and head orientation.

These results confirm that the Dual Focus-3D model performs accurately even in extreme head–gaze conditions, showing its ability to effectively link image features with 3D head vectors. This emphasizes the value of integrating 3D information for reliable gaze estimation.

### 5.3. Cross-Dataset Generalization

We observe variations in extreme gaze angles, head orientations, and the difference between gaze direction and head orientation angles across the datasets.

For the EyeLis dataset, the gaze direction angles range from $\left[-40^{{}^{\circ}},40^{{}^{\circ}}\right]$ for pitch and $\left[-35^{{}^{\circ}},25^{{}^{\circ}}\right]$ for yaw, based on the target positions defined in the dataset (see Section 3). Head orientation varies with pitch values between $\left[-30^{{}^{\circ}},60^{{}^{\circ}}\right]$ and yaw values between $\left[-40^{{}^{\circ}},30^{{}^{\circ}}\right]$.

In the RT-GENE dataset, gaze direction angles range from $\left[-35^{{}^{\circ}},35^{{}^{\circ}}\right]$ for pitch and $\left[-50^{{}^{\circ}},25^{{}^{\circ}}\right]$ for yaw. Two head orientation zones are defined:

- Around pitch $=-170^{{}^{\circ}}$, with yaw between [−0.4,0.8].
- Around pitch $=170^{{}^{\circ}}$, with yaw between [−0.4,0.8].

In the UnityEye dataset, gaze direction angles cover a wider range: $\left[-75^{{}^{\circ}},75^{{}^{\circ}}\right]$ for pitch and $\left[-50^{{}^{\circ}},50^{{}^{\circ}}\right]$ for yaw. Two synthetic head orientation zones were created:

- Around pitch $=-90^{{}^{\circ}}$, with yaw between $\left[-95^{{}^{\circ}},-85^{{}^{\circ}}\right]$.
- Also at pitch $=-90^{{}^{\circ}}$, with yaw between $\left[-35^{{}^{\circ}},-25^{{}^{\circ}}\right]$.

Considering the data coverage for head and gaze angle distributions across all datasets, MPIIFaceGaze is the most suitable for training the model to evaluate its generalization ability. This choice is further motivated by the closer alignment of MPIIFaceGaze’s annotation and acquisition methods with those of other datasets, facilitating superior cross-dataset generalization. Consequently, we used the MPIIFaceGaze dataset for training and evaluated the model on the complete EyeLis, RT-GENE, and UnityEye datasets. As an additional experiment, we also trained the model on RT-GENE and tested it on the full MPIIFaceGaze dataset to further assess its robustness.

The results in Table 5 demonstrate that the Dual Focus-3D model generalizes well, even though the annotation methods and tools differ across datasets. These results confirm that our model remains reliable even under real-world variations in eye appearance and head pose annotations. It is important to highlight that the annotation method employed in EyeLis follows a rigorous procedure combining facial landmark extraction and depth estimation to accurately reconstruct gaze direction within a real-world metric coordinate system, independently of supervised learning. Additionally, an angular error threshold filtering—set, for example, at 2°—ensures that only gaze vectors with guaranteed precision, corresponding to exact fixations, are retained. This careful selection process enables the creation of coherent and usable subsets from a larger dataset, providing EyeLis with significant flexibility to generate datasets tailored to specific precision requirements.

**Figure 6:**
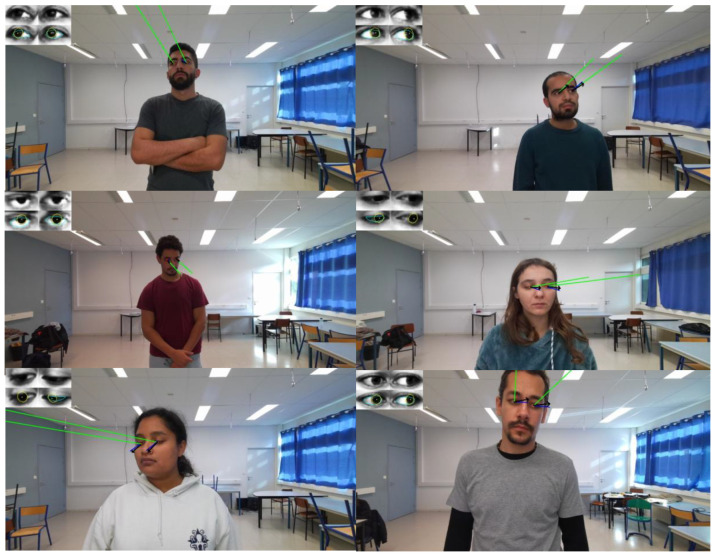
Illustrative results obtained by applying the trained Dual Focus-3D model to evaluate gaze estimation on the EyeLis-DB database, including three vectors: the blue vector represents the ground-truth gaze direction, the green vector represents the ground-truth head orientation, and the black vector represents the predicted gaze direction.

Figure 6 presents qualitative results obtained by applying the trained Dual Focus-3D model to the EyeLis-DB database. Each example includes three vectors: the blue vector is the ground-truth gaze direction, the green vector is the ground-truth head orientation, and the black vector is the predicted gaze direction. All vectors are projected from 3D to 2D using the camera calibration parameters. The results on the test set indicate that the predicted gaze direction closely matches the ground truth, demonstrating the model’s accuracy in estimating gaze based on head orientation and image features.

## 6. Discussion and Limitations

Dual Focus-3D combines eye image features with 3D head orientation vectors to improve accuracy in 3D gaze estimation. The experimental results on both real (EyeLis, MPIIFaceGaze, RT-GENE) and synthetic (UnityEye) datasets show that including head orientation significantly reduces gaze prediction errors, especially in challenging conditions. The model maintains good performance even with large variations in head pose and gaze direction, confirming the importance of head orientation in this task.

However, the method has limitations. It requires both the eye image and the 3D head orientation vector as inputs. Although our results show that head orientation is the most important input, this requirement depends on external hardware for pose capture, which limits the method’s practical deployment.

Another limitation is related to the eye detection method used during preprocessing. We currently use a Haar cascade detector, which is fast but not always reliable under occlusion, blur, or lighting variation. The choice of detector should be adapted to the specific use case, especially when real-time performance is required.

The dual-branch architecture increases computational load, but this remains acceptable with modern hardware. With a suitable system setup, the pipeline can run in real time. For deployment on embedded or low-power devices, further optimization will be necessary.

In future work, we aim to develop an end-to-end model capable of accurately estimating 3D head orientation directly from facial images [50], thereby eliminating the need for external hardware and enhancing system portability. This model will produce precisely annotated 3D head orientation vectors based on well-calibrated facial data.

## 7. Conclusions

In this work, we introduce Dual Focus-3D, a hybrid deep learning framework for accurate 3D gaze estimation in natural environments. The proposed model combines appearance-based eye image features with 3D head orientation vectors using a dual-branch architecture. A residual CNN extracts spatial features from grayscale eye images, while a parallel MLP processes head pose information. These features are fused through a dense integration module optimized for gaze vector regression using an angular loss function. We also introduced EyeLis, a novel dataset containing annotated eye images and corresponding 3D gaze and head pose data. Experimental results on EyeLis and three public benchmarks—MPIIFaceGaze, RT-GENE, and UnityEye—demonstrate that Dual Focus-3D achieves state-of-the-art performance across various conditions. Through cross-dataset analysis, and considering that each dataset presents specific acquisition conditions (e.g., lighting, head orientation, image quality), we demonstrate that our model is robust under diverse real-world scenarios. Furthermore, using EyeLis, we demonstrate that the model is trained with values that closely match the real annotations, exhibiting an angular error deviation of only 2°, which further supports its precision and reliability. We observe that combining visual features with head pose data yields improved accuracy and stability across different datasets, positioning this model as a strong candidate for real-time applications in HCI, AR/VR, and assistive technologies. Future work will explore extending the model to dynamic scenarios and incorporating additional temporal information.

## Figures and Tables

**Table 1:** Comparison of key characteristics across gaze estimation datasets.

Characteristic	MPIIFaceGaze	RT-GENE	UnityEye	EyeLis-DB (Ours)
Acquisition Hardware	Laptop webcam	Kinect v2 (Microsoft Corporation, Redmond, WA, USA) + Motion capture system	Synthetic images generated	Azure Kinect v3 (Microsoft Corporation, Redmond, WA, USA)
Lighting Conditions	Uniform artificial lighting	Natural indoor lighting	Synthetic	Natural indoor or uniform artificial lighting
Subject Count	15	15 (17 sessions)	66,316 different synthetic images	8 (40 sessions)
Acquisition Setup	Controlled laboratory setting	Controlled laboratory setting	Synthetic	Controlled laboratory setting

**Table 2:** Results on the MPIIFaceGaze dataset.

Method	Head	Eye Image	Angular Error (°)
Dilated-Net [43]	Head image	✓	4.42
CA-Net [44]	Head image	✓	4.27
GFNet [45]	Head image	✓	3.96
CTA-Net [46]	Head image	✓	3.91
MAFI-Gaze [29]	Head image	✓	3.84
Dual Focus-3D (Ours)	3D vector	✓	3.24

**Table 3:** Results on the RT-GENE dataset.

Method	Head	Eye Image	Angular Error (°)
I2DNet [47]	Head image	✓	8.40
EG-SIF [48]		✓	7.41
Dual Focus-3D (Ours)	✓	✓	1.73

**Table 4:** Results on the UnityEye dataset.

Method	Head	Eye Image	Angular Error (°)
GazeNet [49]		✓	14.00
UnityEye [19]		✓	9.95
Dual Focus-3D (Ours)	✓	✓	2.39

**Table 5:** Cross-dataset evaluation results.

Training Dataset	Test Dataset	MAE (°)
MPIIFaceGaze [17]	EyeLis [15]	12.22
MPIIFaceGaze [17]	RT-GENE [18]	9.19
MPIIFaceGaze [17]	UnityEye [19]	12.87
RT-GENE [18]	MPIIFaceGaze [17]	8.33

## Data Availability

The dataset supporting the findings of this study, *EyeLis: A 3D Gaze and Head Pose Dataset for Unconstrained Human Interaction, Version 1.0. 2025. Available online: https://im.lis-lab.fr/datasets/EyeLis/V1/ (accessed on 13 May 2025).

## Abbreviations

The following abbreviations are used in this manuscript:

VR	Virtual Reality
CNN	Convolutional Neural Network
LSTM	Long Short-Term Memory
MLP	Multilayer Perceptron
SVR	Support Vector Regression
GPR	Gaussian Process Regression
RNN	Recurrent Neural Network
RGB	Red, Green, Blue
MAE	Mean Angular Error
HCI	Human–Computer Interaction
AR	Augmented Reality
